# Intraoperative localization and protection of important structures of the neck in thyroid surgery

**DOI:** 10.1186/1756-6614-8-S1-A4

**Published:** 2015-06-22

**Authors:** Jan Brzeziński

**Affiliations:** 1 Department of Endocrinology and Metabolic Diseases, Polish Mother’s Memorial Research Institute, Lodz, Poland

## 

Nodular thyroid disease affects thousands of people in Poland. Tumors of the thyroid account for about 1% overall human cancers. Thyroidectomy is the most common surgical operation in endocrine tumors. Operative therapy for benign thyroid nodules is recommended for: progressive increase in nodule size, substernal extension, compressive symptoms of the neck, the development of thyrotoxicosis and patient therapeutic prevalence. In Poland thyroidectomy is the fifth surgical procedure and comprises about 25000 operations yearly. Reduction of surgical injury with simultaneous retention of current safety and radical nature of surgical intervention forces the work into a relatively small operating field. Electric devices enabling the achievement of full and lasting haemostasis during thyroidectomy supplant traditional surgical method (ligature, haemostatic sutures) with no impact on the incidence of perioperative complications, while at the same time allowing to shorten the duration of the procedure. The haemostatic effect is associated with generation of heat, which apart from the intended result may bring about thermal tissue injury. During the surgical procedure it is important to determine the thermal spread around the active tip of electric devices in the operating field during thyroidectomy, and the safe temperature range during the operation to protect important structures of the neck. The mean safe distance of the active tip of an electric device from important anatomic structures is 5mm minimally and depends on the device type, time of operation and its power settings. All the modern techniques of vessel sealing are associated with generation of heat and its spherical spread, which causes thermal injury to the surrounding tissues. Their mode of operation through, among others, structural changes in collagen and elastin, leads to durable connection of sealed vessel walls and tissue structures. These systems enable a safe sealing of vessels of up to 7 mm in diameter.

In conclusions: In the cases analyzed by the author concerning the thyroidectomy techniques, it is recommended to replace electric devices with ligatures or clips or human fibrinogen in place near the laryngeal nerves, parathyroid glands and the trachea. The decision of the change of the method of haemostasis maintenance in the vicinity of crucial structures has been left to the surgeon.

